# Feasibility and acceptability of a peer youth led curriculum to improve HIV knowledge in Northern Tanzania: resilience and intervention experience from the perspective of peer leaders

**DOI:** 10.1186/s12889-021-11876-5

**Published:** 2021-10-23

**Authors:** Kalei R. J. Hosaka, Blandina T. Mmbaga, John A. Gallis, Dorothy E. Dow

**Affiliations:** 1 John A. Burns School of Medicine, University of Hawaii at Manoa, Honolulu, HI USA; 2 Kilimanjaro Christian Medical Centre, Moshi, Tanzania; 3 Kilimanjaro Christian Research Institute, Moshi, Tanzania; 4 Kilimanjaro Christian Medical University College, Moshi, Tanzania; 5 Duke Global Health Institute, Duke University Medical Center, Durham, NC USA; 6 Department of Biostatistics & Bioinformatics, Duke University, Durham, NC USA; 7 Division of Infectious Diseases, Department of Pediatrics, Duke University Medical Center, Durham, NC USA

**Keywords:** HIV, Youth, Peer, Curriculum, Resilience

## Abstract

**Background:**

Youth Living with HIV (YLWH) have reduced adherence to antiretroviral therapy (ART) and worse virologic outcomes compared to children and adults. HIV peer youth led (PYL) interventions contribute to improved retention in care and psychosocial wellbeing. The study objective was to assess the feasibility and acceptability of a PYL HIV curriculum and describe change in participants’ knowledge and impact of leadership on peer leaders’ lives.

**Methods:**

An HIV curriculum was taught during monthly Saturday adolescent HIV clinics at two clinical sites in Moshi, Tanzania (2018–2019). Youth attending clinics were ages 12 to 24 years and received the HIV curriculum during routine clinical care. Peer leaders previously participated in a mental health and life skills intervention called *Sauti ya Vijana* (The Voice of Youth; SYV) and were recommended for leadership by SYV facilitators and clinic staff. Peer leaders were trained and supervised weekly in curriculum delivery using a “train the trainer” model. Data were collected and analyzed using mixed methods. Fidelity checklists were used to measure adherence to the curriculum. Youth participants answered written pre- and post-knowledge questions and evaluated PYL teaching. Semi-structured interviews and the Connor Davidson Resilience scale were conducted with peer leaders before and after assuming the leadership role.

**Results:**

Peer leaders (*N* = 4 male; 3 female) demonstrated high fidelity (96%) to activities in each lesson and participant feedback was positive for curriculum delivery. Participants’ knowledge improved in nine of ten sessions. All but one leader—who moved away before the study ended—demonstrated stable or improved resilience with a mean difference of 3.8 (SD = 7.0) from before the intervention to after assuming the leadership role. Peer leaders reported improved leadership confidence and resilience, and their perception was that the curriculum helped normalize the HIV experience for YLWH attending clinic. Nevertheless, anticipated stigma, difficulty disclosing HIV status, and teaching ability remained barriers.

**Conclusions:**

This study demonstrated that a PYL curriculum to improve HIV knowledge integrated into routine adolescent HIV clinic in Tanzania was feasible, acceptable, and improved knowledge while also benefiting peer leaders, thus providing evidence to continue to support efforts to scale and sustain PYL interventions for YLWH.

**Supplementary Information:**

The online version contains supplementary material available at 10.1186/s12889-021-11876-5.

## Background

There are an estimated four million youth (15–24 years of age) living with HIV globally, 85% of whom live in sub-Saharan Africa [1]. Adolescence is a complicated developmental period, where high priority is placed on fitting into peer groups. Finding acceptance with peers can be tricky in the context of living with HIV, which poses unique social, developmental, and health challenges as a stigmatized and sexually transmitted disease.

Youth living with HIV (YLWH) face numerous psychosocial challenges that impact HIV outcomes, including financial stressors, loss of parent(s), internalized stigma, and difficulty coming to terms with their HIV diagnosis [2–4]. Adequate knowledge and understanding of HIV is associated with engagement in care, adherence to antiretroviral therapy, and treatment outcomes [5, 6]. There is a need for interventions to promote resilience, reduce stigma, improve confidence, and improve HIV knowledge in this population of YLWH [7].

Throughout the HIV pandemic, peer youth led (PYL) interventions have been used in HIV programming to promote behavior change, largely in the context of HIV prevention efforts [8–10]. Because adolescents and youth often learn from and are influenced by peers, it is thought that peer leaders are best able to present health knowledge to encourage behavioral change in youth. Peers often have similar life experiences and may come from the same community [11]. PYL interventions have been shown to contribute to positive change in HIV knowledge, improved community attitudes and norms, and improved adherence, retention in care and psychosocial wellbeing for YLWH [8, 12].

Peer leaders who deliver HIV interventions experience benefit from leadership roles. One group found that peer leaders in a PYL HIV intervention demonstrated improved HIV knowledge and a higher perception of themselves as change agents when compared to control groups [13]. Another group in Rwanda commented on the ways that youth in PYL interventions have gained confidence, competence, and enthusiasm [14].

PYL interventions are not without potential challenges. For instance, peer leaders may not always model best behavior and their peer status may at times conflict with their professional role [15]. Moreover, there may be concern that peers may not impart knowledge as accurately and sensitively as professional counterparts.

There is a need to develop and evaluate PYL interventions for participants living with HIV as well as assess the challenges and effectiveness of PYL interventions in improving HIV outcomes in Tanzania and in other low- and middle-income countries. We designed a 12 session HIV curriculum for youth living with HIV, delivered by peer leaders at two sites in Northern Tanzania. The goal of this study was to assess the feasibility and acceptance of a PYL HIV curriculum integrated into routine clinical care; evaluate change in participants’ knowledge; and to explore resilience, fears, and motivations among peer leaders before and after assuming the leadership role.

## Methods

### Setting

The HIV education curriculum was taught during routine Saturday adolescent HIV clinics held monthly from 2018 to 2019 at two sites in Moshi, Tanzania. The Kilimanjaro Christian Medical Centre (KCMC) is the referral hospital for the Northern zone of Tanzania and began offering an adolescent HIV clinic in 2007 for patients 12–24 years of age. Due to growth in the number of perinatally HIV-infected children becoming adolescents, in 2017, the monthly Saturday clinic was separated into an older group (ages 18 to 24 years) who met on the adult side of the clinic and a younger group (ages 12 to 17 years) who continued to meet on the pediatric side of the clinic. The second site was Mawenzi Regional Referral Hospital (MRRH) Care and Treatment Center located in the center of Moshi, approximately 5 km from KCMC. An adolescent HIV clinic was founded at MRRH in 2014. Similar to KCMC, the MRRH adolescent clinic grew rapidly and in 2017, the clinic was split to care for younger youth (mostly ages 12 to 17) the first Saturday of the month and older youth (mostly ages 18 to 24) the third Saturday of the month.

### Peer leaders and participant characteristics

Peer leaders were 17–24 years of age at the beginning of the teaching period and were recommended for leadership by facilitators of a concurrent mental health intervention and clinic staff. Youth between 12 and 24 years of age receiving ART from adolescent HIV clinics at either KCMC or MRRH and who knew their HIV status were invited to participate. Each month, participants were asked if they were willing to take an anonymous pre-knowledge test before the lesson and a post-knowledge test when the lesson was complete. The forms had a number in the top corner and participants pre- and post-knowledge questionnaires were matched based on form number. Participants were not identified by name or medical record number and those agreeing to participate likely varied each month.

Peer leaders attended weekly training sessions where they reviewed the HIV curriculum content and practiced session delivery in a mock-teaching session the Friday before they taught at the Saturday clinic. The peer leader supervisors, who had experience delivering a youth-focused mental health intervention, took session notes and documented questions that arose during the Friday (practice) and Saturday (live) sessions and provided feedback to peer leaders. Monthly “question and answer sessions” with both the peer leader supervisors and an expert HIV physician allowed peer leaders to further clarify the questions that arose during curriculum sessions. The peer leaders then communicated these clarifications back to youth in the adolescent HIV clinic.

### HIV curriculum

The HIV curriculum was adapted from the HIV curriculum developed by the Baylor International Pediatric AIDS Initiative [16]. Teaching topics are outlined in Table 1. There were twelve lessons in total, ten taught by peer leaders.

**Table 1:** Monthly Lesson Topics^a^

Monthly Lesson Topics	
1. Epidemiology and Pathophysiology of HIV	
2. Clinical Manifestations of HIV	
3. HIV Therapy	
4. Monitoring HIV Infection – CD4 vs viral load	
5. Career Day: Professionals living with HIV share their success stories (not peer-led)	
6. HIV transmission and prevention	
7. Food and Water Safety, Nutrition, and Permaculture	
8. Stigma and Discrimination	
9. ART Adherence	
10. Alcohol and Drugs	
11. Sexual and Reproductive Health (taught by a nurse)	
12. Disclosure of HIV Status	

^a^Adapted with permission from Baylor International Pediatric AIDS Initiative

### Measures

Pre-post knowledge questionnaires for each lesson were designed by peer leaders, supervisors, and local HIV experts. Pre-post knowledge questionnaires included four to five questions per lesson; the post-lesson questionnaire also included three questions about quality of the session delivery and preference for peer leader teaching compared to clinic staff members (see Supplemental File 1). Supervisors documented attendance, punctuality, and fidelity to the curriculum content using detailed fidelity checklists. Fidelity checklists were kept to determine how well peer leaders adhered to the lesson script, which included a list of all the important teaching components in each session as well as questions and responses provided. Supervisors also maintained qualitative session notes from peer leader practice sessions held on Fridays before delivering the content during Saturday clinic.

In-depth semi-structured interviews were conducted with peer leaders before starting their leadership position and again after delivering six curriculum lessons; interview guides were developed specifically for this study (see Supplemental File 2). The Connor Davidson Resilience Scale© (CD-RISC) was administered by the interviewer at the end of each interview and used to quantitatively assess resilience in peer leaders [17]. Interviews were recorded and transcribed in Kiswahili. Interviews were analyzed after being translated into English.

### Quantitative data analysis

Feasibility was assessed using fidelity checklists (percentage score of how closely peer leaders adhered to the lesson script). Acceptability was assessed by mean frequency of responses to a question on each post-lesson questionnaire that assessed preference of peer leader teaching compared to clinic staff members. Baseline peer leader characteristics, pre- and post-intervention peer leader resilience scales, and pre- and post-lesson knowledge assessments of participants were summarized using Stata 16 software (StataCorp, College Station, Texas).

### Qualitative data

An inductive approach was initially used to code and analyze peer leader interviews. Interviews were coded and analyzed in NVIVO. Two researchers independently coded the data. Codes were compiled into a codebook; codes were categorized and sub-categorized. The team relied on dialogical intersubjectivity and group consensus as agreement goals [18].

Thematic analysis was performed to identify themes that addressed our main research question (see Supplemental File 3 for table on generated themes and sub-themes based on thematic analysis) [19]. We used Braun and Clarke’s six-step framework for thematic analysis [20]. Themes were developed deductively from the initial coding process. These themes were reviewed and defined.

### Ethics

Peer leaders provided written informed consent in Kiswahili. Participants answering knowledge questions did not provide written informed consent as no identifying information was collected; IRBs approved the lack of consent taken from participants only answering knowledge questions. The Duke University Medical Center Institutional Review Board (Duke University Protocol ID: Pro00069892), KCMC Research Ethics Committee (No 834, research number 701), and the Tanzanian National Health Research ethics committee of the National Institute for Medical Research (NIMR/HQ/R.8c/Vol.I/1706).

## Results

Characteristics of peer leaders are presented in Table 2. Seven peer leaders (*N* = 4 male; 3 female; 21 years of age on average) participated in the intervention. All peer leaders were perinatally-infected with HIV, and peer leaders had an HIV diagnosis for an average of 11.5 (SD: 0.8) years. As opposed to being told about their HIV status, all peer leaders reported they figured it out on their own before being told by a family member or health care provider.

**Table 2:** Demographics of peer leaders

	*N* = 7
**Age**	
Mean (Standard Deviation)	21.0 (1.0)
Median (Quartile 1, Quartile 3)	20.7 (19.1, 23.8)
Minimum, Maximum	17.9, 24.9
**Sex, male**, n (%)	4 (57%)
**Age at HIV Diagnosis** (per chart; based on date of diagnosis)	
Mean (Standard Deviation)	9.4 (2.8)
Median (Quartile 1, Quartile 3)	9.4 (6.0, 11.6)
Minimum, Maximum	5.9, 13.4
**Socioeconomic status, n (%)**^a^	
House has electricity	4 (57%)
House has indoor plumbing	5 (71%)
Owns a cell phone	5 (71%)
**Highest Level of Educational Attainment, n (%)**	
Less than secondary	3 (43%)
Through secondary	2 (29%)
Vocational	2 (29%)
**Primary Caregiver, n (%)**	
Mom or dad	4 (57%)
Aunt or Uncle	1 (14%)
Grandmother or Grandfather	1 (14%)
Other, unspecified	1 (14%)

^a^Categories are not mutually exclusive

### Feasibility and acceptability

**Fig. 1:**
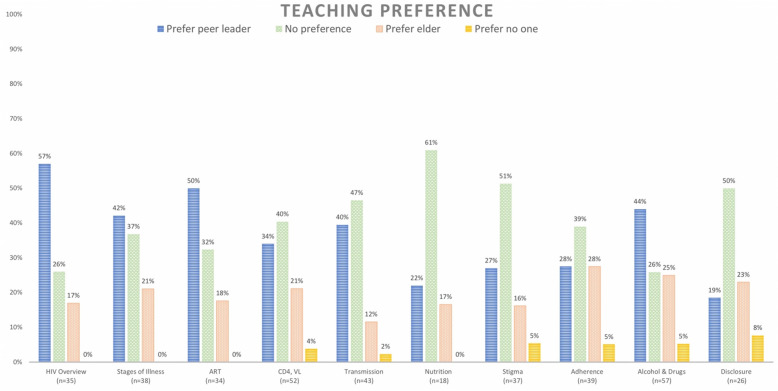
Teaching preference by lesson among participants who took anonymous pre- and post-lesson knowledge assessments

Peer leaders demonstrated good adherence to the protocol, completing on average 96% of activities in lesson plans (range: 82–100%). On average, 39% of anonymized participants across lessons (SD 12%) had no preference for who taught the HIV curriculum. For those who did report a preference, participants preferred peer-led sessions compared to elder or provider-led sessions nearly 2:1 (on average 38% versus 20% in all sessions); 3% of participants preferred that no one teach them. Preference varied by topic (Fig. 1). An average of 85% (range: 71 to 91%) of participants reported learning something new after each session.

### Participant knowledge pre- and post-youth led education sessions

**Fig. 2:**
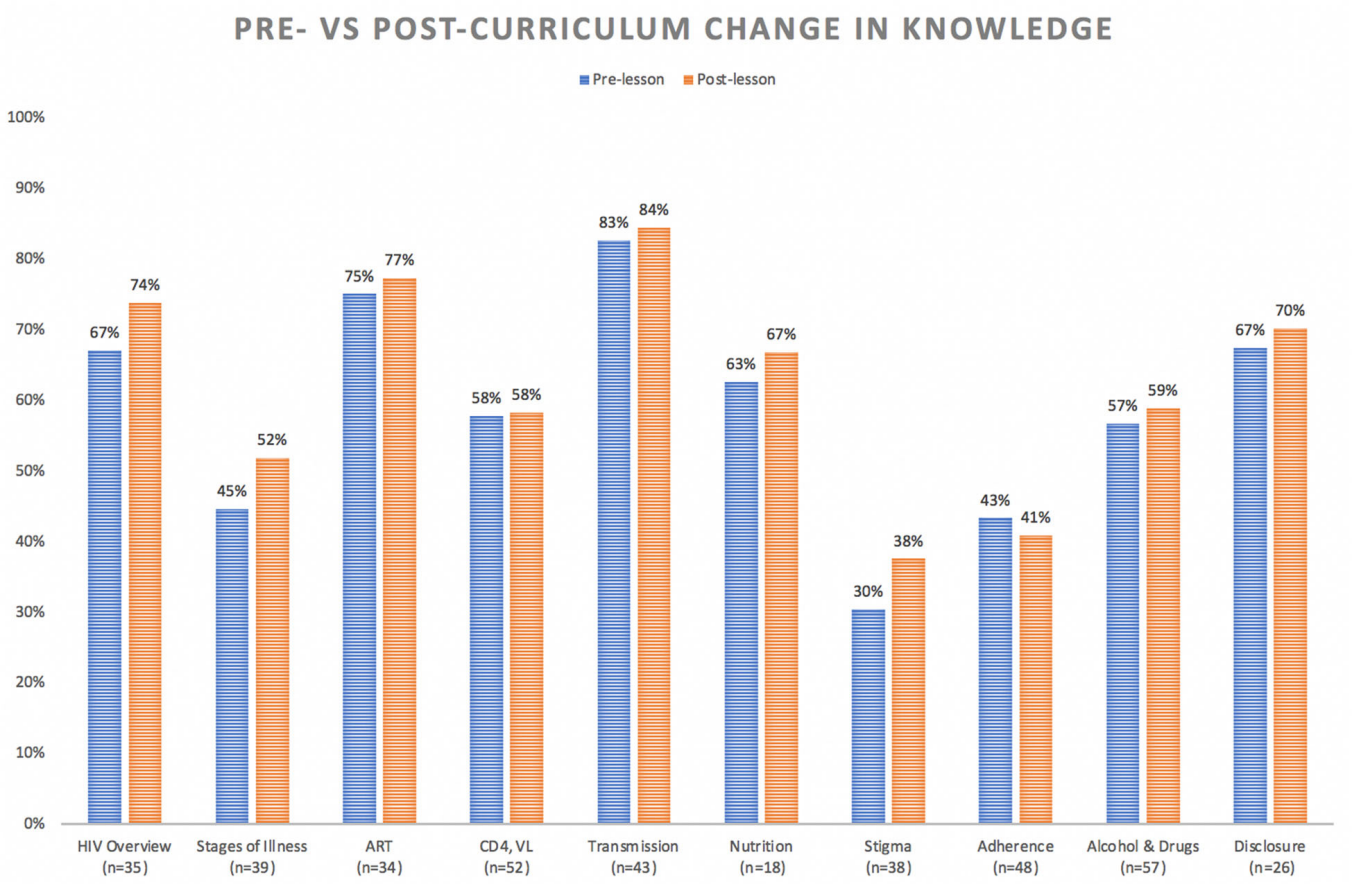
Change in knowledge pre- vs. post-lessons by lesson topic among participants who took anonymous pre- and post-lesson knowledge assessments. ^*^Note: The lesson on nutrition was held at MRRH, but knowledge assessments for that site were misplaced; therefore, the pre- and post-lesson knowledge for this lesson is represented by only KCMC participants. For the lessons that we have differentiated site data entered into the database (“adherence”, “alcohol & drugs”, and “disclosure”) we did not find large differences in pre- and post-knowledge assessment scores between sites (all within 6 percentage points), except for the post-lesson data for the lesson on disclosure (mean overall assessment score post-lesson 74% for KCMC [*N* = 20] vs 58% at MRRH [*N* = 6]). The low N at MRRH is due to this lesson being taught only to the younger youth (ages 12 to 17 years) due to logistical conflicts with the older group (18 to 24 years)

Clinic participants demonstrated knowledge improvement in nine of ten sessions pre- vs. post-intervention (Fig. 2). The first two lessons (HIV overview and Stages of HIV Illness) and the lesson on stigma showed the greatest pre- vs. post-lesson improvement. Topics with a heavier biomedical focus—including principles of antiretroviral therapy, CD4 and viral load monitoring, and adherence (which had a large component of HIV resistance)—showed the least amount of change pre- vs. post-lesson, reflecting that the topic may have been difficult for peer leaders to teach and youth to understand.

### Resilience

**Fig. 3:**
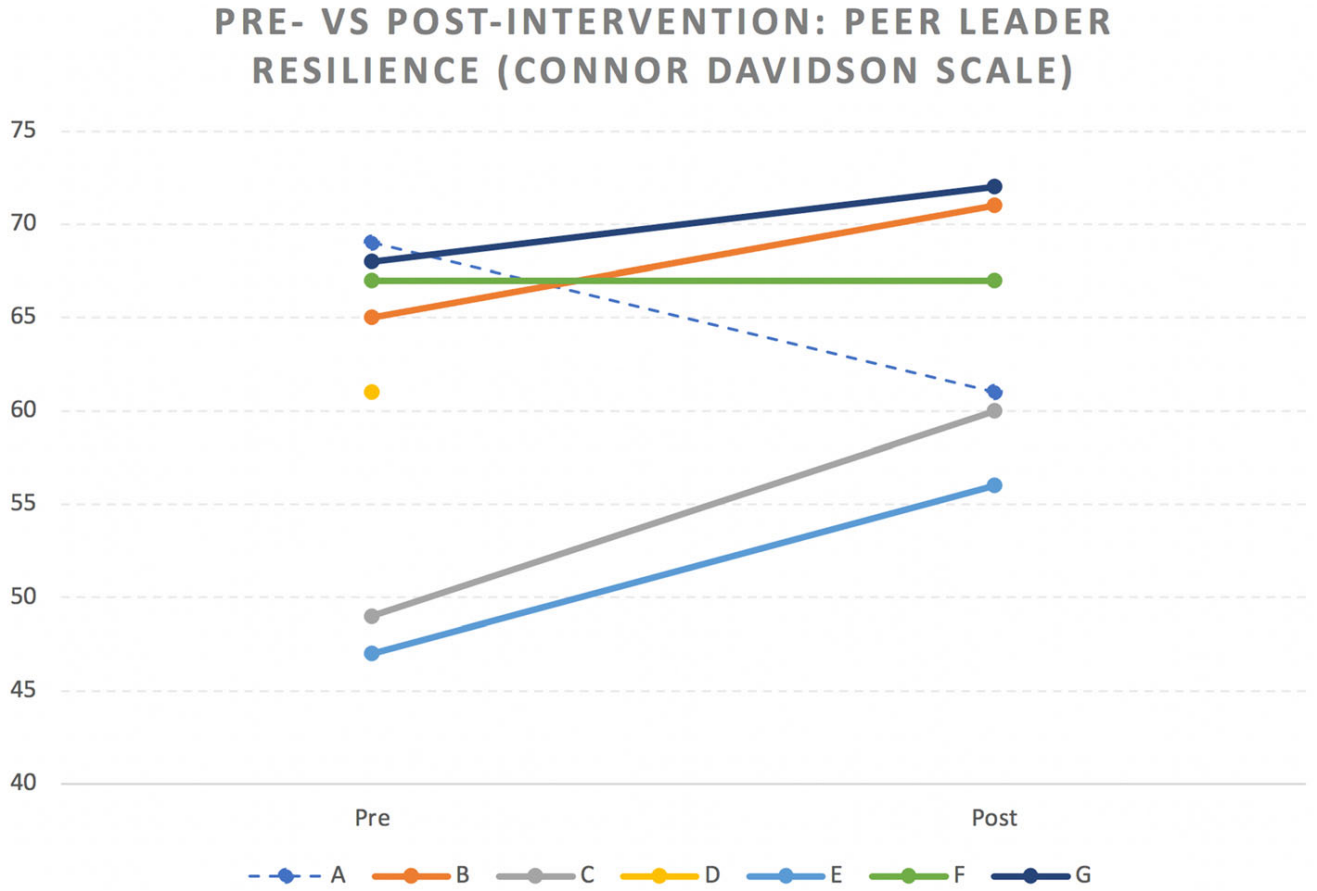
Pre- vs. post-intervention Connor Davidson Resilience scale. *Note:* participant A moved to a new region in Tanzania before the study ended

Peer leaders completed the CD-RISC pre- and post-intervention. All but one leader—who moved away before the study ended—demonstrated stable or improved resilience (Fig. 3), with a mean difference of 3.8 (SD = 7.0; median = 5.5; Q1, Q3 = 0, 9; min, max = − 8, 11). The pre-intervention resilience mean was 60.7 (SD = 9.1; median = 64.0; Q1, Q3 = 49, 68; min, max = 47, 69), while the post-intervention resilience mean was 64.5 (SD = 6.5; median = 64; Q1, Q3 = 60, 71; min, max = 56, 72). One of the seven peer leaders did not have a post measurement due to a recording malfunction during data collection.

### Qualitative thematic analysis: in-depth interviews with peer leaders pre- and post-intervention

#### Importance of peer education in normalizing the HIV experience

Most peer leaders felt that a PYL HIV curriculum had potential to benefit youth. A common theme expressed by peer leaders was that HIV education was key to reducing HIV-associated internalized stigma, including beliefs about disability, mortality, and shame. As a male peer leader explained, “If you have enough education and you know your status the situation of stigmatizing yourself is gone.” Many peer leaders described the ways they struggled with perceived stigma such as believing that they would be isolated by others, rejected by society, and unable to find employment or partners because of HIV; through their leadership they taught youth to see HIV as a chronic disease that they can survive. For example, a male peer leader explained his motivation for being a peer leader: “My goal is to educate people living with HIV.. . so they can know their importance and not give up on life.” Through education, peer leaders taught youth that HIV does not have to be associated with death, isolation, stigma, and discrimination.

Peer leaders felt that youth were more willing to ask questions and more open with them than with adult providers (i.e., nurses, social workers, and physicians) due to shared experience of living with HIV. As a male peer leader explained, “When I teach, youth are free to ask questions.. .. If the nurse teaches, youth are afraid to ask.” The sense among peer leaders was that youth feel more welcomed and supported being taught by a peer who is also living with HIV. Furthermore, peer leaders felt that they can help to normalize the HIV experience for youth. A male peer leader explained: “[Being taught by a peer group leader, youth] will be more knowledgeable and see themselves as normal people.” The hope was that participants would see that it is possible to live a normal and full life with HIV.

#### Fear of discrimination: stigma and HIV disclosure

A recurring theme among peer leaders was a fear of discrimination if others found out about their status. A male peer leader explained that if friends found out about his HIV status, they “will isolate [him] and see he is sick.. .. They will not trust [him] and [he] will not be able to get a partner.” A male peer leader shared: “If they do not have enough education, they may not give me opportunity like jobs and friends may isolate – on the street or at work.” One peer leader explained that his friends who are not living with HIV generally treat him well and with respect; however, he shared: “If [my friends] had known about my [HIV infection], they would despise me and even the friendship would be over, due to poor knowledge. They do not know that an HIV-infected person is a normal person.” Peer leaders expressed a high level of anticipated stigma from the community.

When asked by youth about disclosure, peer leaders unanimously advised they should first trust the person with whom they are considering disclosure as HIV disclosure could have negative consequences on their future in the community. This was true before and after the intervention. A female peer leader shared: “If [you] tell others in society.. .. they can gossip, they can discriminate.” The consensus among peer leaders was that it is important for youth to be careful with disclosing HIV status and only do so with someone who has knowledge about HIV or a person with whom they trust. For youth to be more open with their HIV status, all peer leaders believed education on a community level is needed to reduce stigma.

#### Benefits of leadership

Peer leaders commented on the ways that their confidence and leadership skills improved through the intervention. A female peer leader shared, “Being a peer leader has helped me to become confident. I absolutely believe that you should not lose hope in life. There is today and tomorrow.” Being a peer leader also promoted improved knowledge of peer leaders and encouraged positive behavior change. A male peer leader shared that being a leader improved his adherence to antiretroviral therapy, improved his ability to respond to stress, and reduced his alcohol intake. A female peer leader shared that the leadership experience encouraged her to model good behavior for her peers: “As a peer leader, I should adhere to my medicines so that maybe others can adhere to their medicines [and regularly attend their] clinic date.” There was a sense that for peer leaders to teach effectively, they must lead by example.

#### Challenges of the peer-led intervention

As first-time teachers, peer leaders sometimes found it difficult to engage with the youth and keep their attention. During in-depth interviews, some peer leaders expressed concerns that some in attendance did not pay attention and made jokes. As one female peer leader explained, “Sometimes [those listening] joke as we teach. They see our lesson as not as serious [compared to] nurses or doctors.” Furthermore, certain topics appeared to be more challenging than others to understand. For instance, in session training, some peer leaders had gaps in knowledge about the concept of viral resistance to antiretroviral therapy.

## Discussion

To our knowledge, this is the first study to describe a PYL curriculum integrated into routine HIV clinical care to improve knowledge about HIV specifically for HIV-positive youth. Results from this study support the use of a PYL HIV curriculum integrated into routine adolescent HIV clinics as a feasible and acceptable means to improve HIV knowledge. Peer leaders’ high fidelity to lesson scripts and youth participants’ preference for peer leaders support the feasibility and acceptability of a PYL HIV curriculum, and pre- vs. post-lesson knowledge improved in nine of ten lessons. PYL interventions to date have largely been geared toward HIV prevention efforts [9, 11, 12]; interventions to improve HIV outcomes in YLWH have focused less on improving HIV knowledge per se and more on retention in care and psychosocial wellbeing [8, 14]. Compared to a PYL intervention that showed significant improvement in knowledge about HIV (basic HIV transmission, prevention, and attitudes toward HIV) for in-school (largely HIV-seronegative) adolescents in Nigeria [21], the competency questions within our PYL curriculum were more difficult (see Supplemental File 1). Some lessons showed greater knowledge improvement than others; the fact that topics with a heavier biomedical focus (ART, CD4 and viral load monitoring, and adherence with a component of HIV resistance) showed the least amount of change pre- vs. post-lesson suggests that these lessons may be more effective when co-led by trained clinic staff or require enhanced training of peer leaders.

The PYL HIV Curriculum improved resilience of peer leaders as measured by the CD-RISC (developed in the United States) by a mean of 3.8 points. Resilience can be understood as the ability or resolve of an individual to cope with stress [17, 22]. Peer leader resilience scores (mean 60.7 pre-intervention and 64.5 post-intervention) are below standard reference scores (mean 80.4, SD 12.8) seen in the CD-RISC US adult “general population” (i.e., non-help seeking, 77.4% white-American, mean age of 43.8); however, they are consistent with scores in adolescents in South Africa and numerous other cohorts globally that include students and young adults [17, 23, 24]. The fact that a wide range of CD-RISC scores have been reported in the literature supports the notion that resilience is influenced by culture, age, and other factors [25]. Identifying and harnessing resilience in YLWH can have important benefits on both personal and socioecological levels [26]. All but one peer leader demonstrated improved or stable resilience over the course of the intervention; one peer leader did not have a recorded post-intervention resilience measure due to a problem with the audio recording process. The peer leader who saw a decrease in resilience moved to a new city during the intervention but conducted her post-interview by phone; her drop in resilience in some ways could represent how resilience in youth may be dependent on social networks (i.e., removing supportive social networks can negatively impact resilience). Although it is difficult to determine whether a mean difference of 3.8 is clinically meaningful, qualitative thematic analysis suggests that peer leaders gained confidence in their leadership skills and ability to cope with stress, which are components of resilience; peer leaders also saw themselves as examples of leadership [15].

While the PYL HIV curriculum normalized the HIV experience and improved leadership confidence among peer leaders, anticipated stigma and concerns regarding HIV status disclosure remained barriers. Interventions that normalize HIV and facilitate peer support have been shown to be effective in reducing HIV-related stigma and improving mental wellbeing [27–29]. Peer leaders’ anticipated stigma and fear of disclosure is an example of the ways in which stigma among perinatally-infected YLWH remains prevalent [4, 30]; this is despite widespread rollout of antiretroviral therapy, which has been associated with reduced stigma among adults living with HIV in sub-Saharan Africa [27, 31]. Compared to what is seen in adult populations, HIV-related stigma for YLWH is complex due to developmental, sociocultural, and economic challenges that come with HIV for adolescents and young adults [3, 32]; further research is needed to better understand stigma in this population. Moreover, to create lasting change, interventions should understand and address anticipated stigma and concerns regarding HIV status disclosure in addition to improving HIV knowledge. Nevertheless, how to best address anticipated sigma and disclosure concerns for YLWH in contexts such as Northern Tanzania remains unclear and is a challenge for researchers and practitioners alike.

The study has several limitations. Pre- and post-knowledge assessments were collaboratively designed by peer leaders, supervisors, and HIV experts. They were not validated nor adjusted despite the variety of participant ages (12–24 years of age). There were limitations on data capture and data entry that precluded robust analysis between sites. PYL teaching was integrated into routine clinic and participants could be called to see the health care provider during the lesson. As such, some pre- and post-lessons assessments were possibly completed by different people or in groups. Teaching is a skill that is refined over many years. Peer leaders had varying levels of teaching ability despite intensive weekly training. Peer to peer interventions require thoughtful peer leader selection and ongoing and intensive supervision. Relocation of a highly trained female peer leader interrupted the group dynamic and left only male peer leaders at the MRRH clinic. Education level of a peer leaders may have been a limiting factor in the teaching. Finally, low sample size of peer leaders limited resilience analyses.

## Conclusion

This study demonstrates that a PYL curriculum to improve HIV knowledge integrated into routine adolescent HIV clinic in Tanzania is feasible, acceptable, and may improve knowledge while also benefiting peer leaders, thereby supporting efforts to scale and sustain PYL interventions for YLWH.

## Supplementary Information

**Additional file 1.** Peer Youth Led HIV Curriculum Knowledge Assessments.

**Additional file 2.** Peer Leader In-Depth Interview Guide.

**Additional file 3.** Generated themes and sub-themes based on thematic analysis.

## Data Availability

DTA file used for all quantitative analyses will be made available upon request. A dofile was created in *STATA*. The code will be made available upon request to Dorothy Dow (dorothy.dow@duke.edu).

## Abbreviations

YLWH: Youth living with HIV
PYL: Peer Youth Led
KCMC: Kilimanjaro Christian Medical Centre
MRRH: Mawenzi Regional Referral Hospital
CD-RISC: Connor Davidson Resilience Scale©
