# Supplementary material for: Feasibility and acceptability of a peer youth led curriculum to improve HIV knowledge in Northern Tanzania: resilience and intervention experience from the perspective of peer leaders
Source: BMC Public Health. 2021 Oct 23;21:1925. doi: 10.1186/s12889-021-11876-5 (PMC8542314; doi:10.1186/s12889-021-11876-5)
Supplement: Supplementary file 1 — Additional file 1. Peer Youth Led HIV Curriculum Knowledge Assessments. [file 12889_2021_11876_MOESM1_ESM.docx]

Supplemental Document 1. Peer Youth Led HIV Curriculum Knowledge Assessments

Note:

- *Correct answers are in* ***bold***
- *Questions where more than 75% of assessments (pre or post) did not have the correct answer are highlighted*

Lesson 1: Epidemiology and Pathophysiology of HIV:

1. AIDS is the virus that causes HIV
   1. True
   2. **False**
2. How long does HIV stay in the body?
   1. If ARV medication is taken correctly you can be cured within 6 months
   2. If ARV medication is taken correctly you can be cured within 1 year
   3. **HIV stays in the body forever**
3. How does HIV attack your immune system?
   1. **Attacks CD4 cells and makes more copies of itself**
   2. Attacks red blood cells and makes more copies of itself
   3. Floats in the blood and makes more copies of itself
4. When is a person most likely to pass HIV to another person?
   1. Late infection when viral load is low
   2. **Early stages of infection when viral load is high**
   3. Person is very likely to pass on HIV even if they are on medication and regardless of viral load
5. Why do children and babies who have HIV react differently than adults with HIV?
   1. They do not have CD4 cells
   2. **They have immature CD4 cells**
   3. They react the same as adults

Lesson 2: Clinical Manifestations of HIV:

1. What happens during the first phase of HIV infection?
   1. Person becomes infected with opportunistic infections
   2. Person begins to lose weight, feel sick, may have headaches
   3. **Person feels well since their body is able to fight off the infection**
2. What happens during the second phase of HIV Infection?
   1. CD4 (askari) cells are able to fight off HIV and an infected person feels well
   2. **CD4 (askari) cells begin to die as HIV gets stronger and the infected person starts to feel sick**
   3. CD4 (askari) cell numbers are very low and the person might get sick with opportunistic infections
3. What are examples of common opportunistic infections?
   1. **Tuberculosis** (72%)
   2. Heart disease
   3. Malaria
   4. Chickenpox
4. What happens if you are on ART?
   1. Decreases CD4 (askari) levels in your body
   2. **ART keeps your immune system strong and prevents progression of HIV**
   3. Cures HIV
5. How is your body affected as more CD4 (askari) die?
   1. Immune system weakens
   2. Feel very tired and begin to lose weight
   3. Become sick with infections the immune system was able to prevent before
   4. **All of the above**

Lesson 3: HIV Therapy:

1. How does antiretroviral therapy work to fight HIV?
   1. **Makes the virus sleep so it doesn’t make more copies of the virus**
   2. Kill all the virus and cure the body of HIV
   3. Increase the number of red blood cells so you don’t get anemia
2. How can a doctor know if you’re taking antiretroviral therapy like you are supposed to?
   1. Viral load increases
   2. **Viral load not detectable or less than 200 copies/mL**
   3. Viral load remains detectable
3. If someone is taking their antiretroviral therapy regularly then they do not have to worry about transmission to someone else.
   1. True, because it is impossible to have a resistant virus
   2. **False, because my virus might be resistant to medication and still be detected in the blood**
4. If you take ART long enough you will be cured of HIV.
   1. True
   2. **False**

Lesson 4: Monitoring HIV Infection – CD4 vs. Viral Load

1. What is viral load?
   1. **A blood test that measures the number of copies of HIV in the blood**
   2. A measure of CD4 count (or number of soldiers in the blood)
   3. Measure of red blood cell count
2. Having a low CD4 count is good.
   1. True, because CD4 (soldiers in the blood) can be harmful
   2. **False, because CD4 (soldiers in the blood) help fight infection**
3. What are opportunistic infections?
   1. Infections that take advantage of a weak immune system
   2. Infections that normally do not occur in healthy individuals
   3. Infections that usually are the cause of death for individuals living with AIDS
   4. **All of the above**
4. An undetectable viral load means you no longer have HIV.
   1. True, because the medication removed HIV from my body
   2. **False, because the medication put the virus to sleep, but it can still wake up**

*Note: Lesson 5: Career Day: Professionals living with HIV share their success stories (not peer-led)*

Lesson 6: HIV Transmission and Prevention:

1. Circle the answer that correctly identifies 4 ways HIV can be spread to another person.
   1. Sweat, urine, blood, semen
   2. Urine, sweat, tears, breast milk
   3. **Blood, semen, breast milk, vaginal fluids**
   4. Semen, urine, tears, breastmilk
2. Two people who are HIV+ **do not** need to use a condom when engaging in sex.
   1. True, because both people already have HIV and condoms don’t protect against other infections or pregnancy
   2. **False, because people need to protect against other infections or pregnancy**
   3. True, because condoms can give you HIV
3. HIV can be transmitted even if it’s the first time you’re having sex.
   1. **True**
   2. False
4. If one partner has HIV and the other partner doesn’t (serodiscordant), circle the best way to prevent HIV transmission.
   1. **Use condoms and be fully adherent to antiretroviral therapy**
   2. Avoid penetrative vaginal, but anal sex or oral sex are ok (no risk of transmission)
   3. There is no way to prevent HIV transmission

Lesson 7: Food and Water Safety, Nutrition, and Permaculture:

1. Why is it important to eat your vegetables?
   1. Contains more protein than animal foods
   2. **Helps protect you from disease and the vitamins help you to digest your food well**
   3. Only important before puberty, not once you’re done growing
2. How does **poor** nutrition effect HIV?
   1. Has no effect on an HIV+ person
   2. **Weakens the immune system**
   3. Gives you more energy and strength to boost your immune system
3. What is an example of permaculture?
   1. Planting flowers around your house to make your area more beautfiul
   2. Eating out at restaurants to help local businesses
   3. **Way of living that uses most of our resources to provide a more permanent food source**
4. What can you do to practice good food and water safety?
   1. **Wash hands properly**
   2. Save water by not washing fruits and vegetables
   3. Store fresh produce next to meat to help them stay fresh longer
   4. Avoid eating fresh foods and only eat sweets/chips

Lesson 8: Stigma and Discrimination:

1. Which one of these might cause stigma?
   1. Someone looking sick (having a rash that is very noticeable)
   2. Lack of knowledge about a disease
   3. Fear of getting HIV
   4. **All of the above**
2. Discrimination is an attitude or belief while stigma is an action.
   1. True
   2. **False**
3. Who can contribute to stigma?
   1. Family members
   2. You yourself
   3. Members of your community
   4. Friends
   5. **All of the above**
4. What is an example of secondary stigma?
   1. An orphan of HIV positive mother/father is stigmatized against
   2. **Stigmatizing yourself because of your HIV status**
   3. Sex worker or street child being stigmatized
   4. Having an HIV child stay in a separate bedroom

Lesson 9: ART Adherence:

1. What is adherence?
   1. Taking your medication the way it was prescribed 50% of the time
   2. Not taking your medication correctly causing your virus to wake up
   3. Developing resistance to ART
   4. **Taking your medication (like ART) at the correct time each day**
2. How many doses can you miss before risking resistance to treatment? (*in hindsight: poorly worded question*)
   1. One dose per week
   2. One dose per month
   3. One dose per day
   4. **Resistance takes a long time to develop even if you miss doses regularly**
3. What happens when the HIV virus develops resistance
   1. Virus makes small changes to itself (mutates)
   2. Virus stays awake even if you take ART as instructed
   3. Virus can only go back to sleep if a different ART medication is started (2^nd^ line)
   4. **All of the above**
4. If you missed a dose of your medication you should wait until the next day to take double the dose.
   1. True (there is no difference if you take it the same day or the next day)
   2. **False (better to take it as soon as possible)**
5. HIV virus can still develop resistance even if you have good adherence
   1. **True (random mutations over a long period of time can occur)**
   2. False

Lesson 10: Alcohol and Drugs:

1. Which of the following are affected by drinking alcohol?
   1. Judgement
   2. Coordination
   3. Vision
   4. Balance
   5. **All of the above**
2. Why is drinking alcohol dangerous for adolescents?
   1. Alcohol increases good connections in the brain
   2. **Your brain is still maturing, and alcohol can cause changes to your brain**
   3. Drinking alcohol is fine even if you are very young and you are still under adult supervision
   4. Alcohol is only dangerous to adolescents who drink everyday
3. What is true about Fetal Alcohol syndrome?
   1. **Causes damage to a baby’s brain and body when a pregnant woman drinks alcohol**
   2. Is curable after birth
   3. Can only be caused by mothers who are heavy drinkers (drink 7 days/week)
4. The impact of alcohol on an adolescent’s brain is very small and is easily treated
   1. True
   2. **False**

*Note Lesson 11: Sexual and Reproductive Health (taught by a nurse, not peer-led)*

Lesson 12: Disclosure of HIV Status:

1. What is disclosure?
   1. **When a person is informed of their own HIV status or informs someone else about their HIV status**
   2. Act of hiding your HIV status from your friends and family
   3. Being treated differently once your HIV status becomes known
2. What is an advantage of disclosure?
   1. People might treat you differently
   2. People might be untrustworthy and tell others
   3. **You can talk freely with this person about any feelings you have about living with HIV**
   4. Someone might not want to be your friend anymore
3. Why might it be important to disclose your status to your romantic partner?
   1. To prevent transmission
   2. You can seek counseling and testing together
   3. Prevent transmission to child if an HIV+ woman gets pregnant
   4. They can help you with emotional support and other assistance
   5. **All of the above**
4. Telling your service provider about your HIV status is important because it helps you access support and information about HIV prevention
   1. **True**
   2. False

3 Questions Included In Each Post-Knowledge Questionnaire:

1. Compared to the usual teaching during Teen Club, how did you like being taught by a peer?
   1. I prefer being taught by an elder (nurse or social worker like usual)
   2. I prefer being taught by a youth (the peer youth leader like today)
   3. I don’t like being taught by anyone
   4. I don’t have a preference for elder or youth as the teacher. Either is ok.
2. Compared to the usual teaching during Teen Club, how much did you learn about HIV that will help you in your daily life?
   1. I didn’t learn much today because of the peer youth leader (wasn’t a good teacher/elder teachers are better)
   2. I didn’t learn much today because I already knew all the information (didn’t matter if it was a peer youth leader or an elder)
   3. I learned a lot today because of the style of teaching by the peer youth leader
3. Any other comments you have about this session? ­­­­­­­­__________________________________________________________________________________________________________________________________________________________________________________________________________________________________________
