# Supplementary material for: Feasibility and acceptability of a peer youth led curriculum to improve HIV knowledge in Northern Tanzania: resilience and intervention experience from the perspective of peer leaders
Source: BMC Public Health. 2021 Oct 23;21:1925. doi: 10.1186/s12889-021-11876-5 (PMC8542314; doi:10.1186/s12889-021-11876-5)
Supplement: Supplementary file 2 — Additional file 2. Peer Leader In-Depth Interview Guide. [file 12889_2021_11876_MOESM2_ESM.docx]

**Supplemental Document 2**

In-Depth Interview Guide Part 1

ESTABLISHING RAPPORT/EXPLORING SELF PERCEPTIONS

1. Tell me a little about yourself.
2. What is important to you in your life?
3. Why is it important to you?
4. What is something that motivates you each day?
5. How do other people describe you?
6. How do you describe yourself?

LIVING SITUATION AND SOCIOECONOMIC STATUS

1. Now I’d like to hear about your living situation.
2. Where do you live?
3. Who do you live with?
4. Does your house have plumbing? Electricity?

Relationships

1. How is your relationship with your caregiver? (Probes: aware of your status, treat you differently than others in the households)
2. How open are you with your caregiver? Do you feel comfortable talking about everything with them?
3. Tell me about your close friends. (Probes: aware of status)
4. Describe your ideal secure and open friendship.
5. How supportive or unsupportive are your current friends?
6. How aware are your friends in your role as a peer group leader?
7. How often do you turn to your friends when you are feeling stressed or overwhelmed?
8. How capable are your friends at being able to reduce your stress?
9. Describe your current romantic relationship. (Probe: Is your boyfriend/girlfriend/partner aware of your HIV+ status, how long have you been together)
10. What is your boyfriend/girlfriend/partner’s HIV status?
11. Have you ever turned to your partner when you were stressed? Were they a good resource?
12. Describe your ideal supportive relationship.
13. How supportive or unsupportive is your current partner?
14. How open are you in your current relationship? (Probes: talk about your status, stress, negative events)

Teen Club – Now I would like to talk about Teen Club.

1. When did you start attending Teen Club? (Probes: How many years has it been)
2. What made you start coming to Teen Club?
3. What kind of education have you received during Teen Club?
4. Who taught the education?
5. What did you like about it?
6. What did you not like about it?
7. How different do you think it will be with peer leaders teaching as opposed to the previous structure?

Being a Peer Leader

1. What is your primary reason for becoming a peer group leader?
2. How do you feel about leading your first lesson? (Probes: nervous, anxious, excited)
3. What do you think makes you a good leader?
4. How do you plan to make participants comfortable during the lessons? (Comfortable enough to ask questions and engage)
5. How have you prepared to lead your first lesson?
6. Have you spent time outside of training?
7. How do you think being a peer group leader will help you in your future? (Probes: disclosure, explaining HIV transmission to someone, career opportunity or skill)
8. What worries you about being a peer group leader?
9. What excites you about being a peer group leader?

Stress and Coping

1. How well do you cope in stressful situations?
2. How often do you feel overwhelmed by stress?
3. What do you try to do in order to reduce stress?
4. How often do you feel overwhelmed with your treatment or HIV status?
5. When something bad happens how do you cope with that negative event?

Disclosure and Stigma

1. Now I want to talk about living with HIV.
2. Who have you disclosed your status to?
3. Have you had any bad experiences telling anyone? Describe the situation.
4. How supportive is your immediate family? (caretakers, siblings, etc.)
5. Why do you think you haven’t disclosed your status to others? (Probes: why they haven’t disclosed to – friends, other family)
6. How do you think people will treat you if they become aware of your status?
7. Describe a time you felt you were treated differently because of your status.
8. Do you think having HIV limits you? (Probes: career, relationships)
9. Why do you think people treat those who have HIV differently? (Probes: lack of education, religious beliefs, e.g. divine justice, cultural beliefs)
10. What would you like to tell them about HIV to get them to change their minds?
11. What do you think needs to happen to reduce the stigma around HIV in your community?
12. If one of the youth came to you asking for advice on how to disclose their status to someone, what would you tell them?

Connor Davidson Resilience Scale © (CD-RISC)

CLOSING OF INTERVIEW

1. What else haven’t I asked you that you think I should know?
2. I would like to thank you for sharing your experiences with me today. Is there anything else before we conclude the interview?
3. If I have any other questions would you be willing to discuss it with me?

In-Depth Interview Guide Part 2

ESTABLISHING RAPPORT/EXPLORING SELF PERCEPTIONS

1. Tell me a little about yourself.
2. What is important to you in your life?
3. Why is it important to you?
4. What motivates you to take your medicine every day and to be adherent?
5. How do you describe yourself as a peer group leader?

Relationships

1. Describe any changes (if any) to your relationships since becoming a peer group leader? (Probes: relationship with caregiver, friends); Specifically with other PGL
2. How do you think knowing your friend’s HIV status (or them knowing yours) impacts how open you are around them?
3. How supportive or unsupportive are your current friends? (Probe: How would it change if they knew your HIV status)
4. How aware are your friends in your role as a peer group leader? (Probes: have they noticed any changes/made comments)
5. How aware is your family in your role as a peer group leader? (Probes: have they noticed any changes/made comments)

Being a Peer Leader

1. What do you think makes you a good leader?
2. What do you think you’ve learned about yourself after leading Teen Club education sessions?
3. How has your opinion on peer group leaders changed?
4. How do you feel the youth see peer group leaders? (Probes: compared to nurses or social workers providing education)
5. If you could go back and give yourself advice before you started leading what would you change?
6. What is your favorite aspect of being a peer leader? Least favorite?
7. How has training helped/hurt you in your life?
8. After leading a couple of lessons what are you going to change about your teaching method moving forward?
9. How comfortable are the youth in asking you questions and engaging in discussion?
10. How is using peer group leaders to teach different than before (when nurses taught)?
11. Describe how you deal with youth who are disruptive during Teen Club. (Probes: youth that make jokes, ask questions they know the answers to)
12. How close do you follow the lesson plan while teaching?
13. Have there been any times you had to change anything through the course of the lesson? Please describe the situation.
14. How receptive are the youth while you’re teaching? (Probes: eye contact, asking questions)
15. What do you think of the previous structure for the lessons compared to now?
16. What do you like about the new structure?
17. What do you not like about it?
18. What suggestions do you have to make this teaching model better?

Stress and Coping

1. How well do you cope in stressful situations?
2. How often do you feel overwhelmed by stress?
3. What do you try to do in order to reduce stress?
4. How often do you feel overwhelmed with your treatment or HIV status?
5. When something bad happens how do you cope with that negative event?
6. How has teaching changed the way you cope with stress if any?

Disclosure and Stigma

1. Now I want to talk about living with HIV.
2. Who have you disclosed your status to? Has this changed since beginning PGL training?
3. Have you had any bad experiences telling anyone? Describe the situation.
4. How supportive is your immediate family? (caretakers, siblings, etc.)
5. Why do you think you haven’t disclosed your status to others? (Probes: why they haven’t disclosed to (friends, other family))
6. How do you think people will treat you if they become aware of your status?
7. Describe a time you felt you were treated differently because of your status.
8. Do you think having HIV limits you? (Probes: career, relationships)
9. Why do you think people treat those who have HIV differently? (Probes: lack of education, religious beliefs (divine justice), cultural beliefs)
10. What would you like to tell them about HIV to get them to change their minds?
11. How important is education about HIV to you?
12. What do you think needs to happen to reduce the stigma around HIV in your community?
13. What has this experience of teaching peers taught you about educating others about HIV? (Probes: Do they feel comfortable going into their community to teach?)
14. Do you feel you have any change in HIV related stigma? (Probes: from the community, from yourself (internal stigma))
15. If one of the youth came to you asking for advice on how to disclose their status to someone, what would you tell them?

Connor Davidson Resilience Scale © (CD-RISC)

CLOSING OF INTERVIEW

1. What else haven’t I asked you that you think I should know?
2. I would like to thank you for sharing your experiences with me today. Is there anything else before we conclude the interview?
3. If I have any other questions would you be willing to discuss it with me?
