# Supplementary material for: Feasibility and acceptability of a peer youth led curriculum to improve HIV knowledge in Northern Tanzania: resilience and intervention experience from the perspective of peer leaders
Source: BMC Public Health. 2021 Oct 23;21:1925. doi: 10.1186/s12889-021-11876-5 (PMC8542314; doi:10.1186/s12889-021-11876-5)
Supplement: Supplementary file 3 — Additional file 3. Generated themes and sub-themes based on thematic analysis. [file 12889_2021_11876_MOESM3_ESM.docx]

Supplemental File 3. Generated themes and sub-themes based on thematic analysis

| Theme/sub-theme | Typical examples or quotes |
| --- | --- |
| Theme 1. Importance of Peer Education in Normalizing the HIV Experience | |
| 1a. Education | “If you are living with a person who lacks education, he or she stigmatizes you.” |
| 1b. Educating self [re: HIV] | “HIV education is very important to me in order to help myself and to see I have value more than [HIV], in good adherence, to remember clinic date always.” |
| 1b. Peer-Led Intervention | “[Fellow youth] were not comfortable to ask a nurse questions that they ask me, so if they were with peer leaders, they are free to talk because they (peer leaders) are the same age or youth like them.” |
| 1d. Normalization of HIV | “[Being taught by a peer leader] they will be more knowledgeable and see themselves as normal people.” |
| Theme 2. Fear of Discrimination: Stigma and HIV Disclosure | |
| 2a. Fear of discrimination | “[If my friend found out about HIV] they would no longer have them their friend again. It would have been very hard to accept maybe the isolation because they know I have HIV infection.” |
| 2b. Disclosure | “I think if I tell others [about HIV] they bring me to problem the society, they can gossip, they can discriminate like that.” |
| 2c. Self-stigma | “Very few times [in the past] for example I said if I have HIV infection I’m not supposed to play with my fellows, so I stigmatized myself.” |
| Theme 3. Benefits of Leadership |  |
| 3a. Confidence | “To be a peer leader has helped me to become confident. I absolutely believe in myself that you should not lose hope in life – there is today and tomorrow.” |
| 3b. Set an Example | “As a peer leader, I should adhere to my medicines so that maybe others can adhere to their medicines [and regularly attend their] clinic date.” |
| Theme 4. Challenges of the peer led intervention | “Sometimes they joke as we teach. They see our lesson as not as serious [compared to] nurses or doctors.” |
